# Impact of Bolus Dosing versus Continuous Infusion of Piperacillin and Tazobactam on the Development of Antimicrobial Resistance in Pseudomonas aeruginosa

**DOI:** 10.1128/AAC.00867-13

**Published:** 2013-12

**Authors:** T. W. Felton, J. Goodwin, L. O'Connor, A. Sharp, L. Gregson, J. Livermore, S. J. Howard, M. N. Neely, W. W. Hope

**Affiliations:** The University of Manchester, Manchester Academic Health Science Centre, NIHR Translational Research Facility in Respiratory Medicine, University Hospital of South Manchester NHS Foundation Trust, Manchester, United Kingdoma; Antimicrobial Pharmacodynamics and Therapeutics, Department of Molecular and Clinical Pharmacology, University of Liverpool, Liverpool, United Kingdomb; Laboratory of Applied Pharmacokinetics, University of Southern California, School of Medicine, Los Angeles, California, USAc

## Abstract

Management of nosocomial pneumonia is frequently complicated by bacterial resistance. Extended infusions of beta-lactams are increasingly being used to improve clinical outcomes. However, the impact of this strategy on the emergence of antimicrobial resistance is not known. A hollow-fiber infection model with Pseudomonas aeruginosa (PAO1) was used. Pharmacokinetic (PK) profiles of piperacillin-tazobactam similar to those in humans were simulated over 5 days. Three dosages of piperacillin-tazobactam were administered over 0.5 h or 4 h, with redosing every 8 h. Two initial bacterial densities were investigated (∼10^4^ CFU/ml and ∼10^7^ CFU/ml). The time courses of the total bacterial population and the resistant subpopulation were determined. All data were described using a mathematical model, which was then used to define the relationship between drug concentrations, bacterial killing, and emergence of piperacillin resistance. There was logarithmic growth in controls in the initial 24 h, reaching a plateau of ∼9 log_10_ CFU/ml. Bacterial killing following administration of piperacillin via bolus dosing and that after extended infusions were similar. For the lower initial bacterial density, trough total plasma piperacillin concentration/MIC ratios of 3.4 and 10.4 for bolus and extended-infusion regimens, respectively, were able to suppress the emergence of piperacillin resistance. For the higher initial bacterial density, all regimens were associated with progressive growth of a resistant subpopulation. A stratified approach, according to bacterial density, is required to treat patients with nosocomial pneumonia. Antimicrobial monotherapy may be sufficient for some patients. However, for patients with a high bacterial burden, alternative therapeutic strategies are required to maximize bacterial killing and prevent antimicrobial resistance.

## INTRODUCTION

The attributable mortality of hospital-acquired pneumonia (HAP) and ventilator-associated pneumonia (VAP) remains high despite treatment with antimicrobial chemotherapy (1). Pseudomonas aeruginosa is a common cause of nosocomial pneumonia (2). Approximately 10 to 50% of patients treated for nosocomial pneumonia develop antimicrobial resistance (3, 4). The development of resistance in P. aeruginosa may account for a proportion of clinical failures following administration of standard therapeutic regimens. Emergence of antimicrobial resistance compromises the outcome of individual patients but also has significant ramifications for the treatment of critically ill populations (4, 5).

For β-lactam antibiotics, the pharmacodynamic index that best links drug exposure with the antibacterial effect is the fraction of the dosing interval during which the free drug concentrations are above the bacterial MIC (f*T* > MIC) (6, 7). Use of extended or continuous infusion maximizes the time during which drug concentrations are above the MIC (8, 9). Compared with bolus dosing, increased bacterial killing is seen both *in vitro* and *in vivo* with infusions of β-lactam antibiotics (10–12). *In silico* models suggest that exposures produced by infusions of β-lactam antibiotics generate a greater probability of target attainment than those attained with bolus dosing (13). Despite this, few studies have demonstrated a clinical advantage associated with β-lactam infusions, and there is no information on the use of such regimens and the likelihood of emergence of drug resistance (14, 15).

The pharmacodynamic index that best links drug exposure and the emergence of antimicrobial resistance is poorly defined. For meropenem, the ratio of minimum concentration to MIC (*C*_min_/MIC) has been linked to suppression of resistance (16). Studies using *in vitro* models suggest that emergence of resistance often follows an “inverted U” pattern—in this case, there is no amplification of resistant subpopulations at either low or high drug concentrations (17). Here, we used an *in vitro* hollow-fiber infection model (HFIM), with Pseudomonas aeruginosa, to examine the impact of administering piperacillin-tazobactam by bolus dosing and extended infusion on the emergence of piperacillin resistance.

## MATERIALS AND METHODS

### Antimicrobial agent.

For the HFIM, the clinical formulation of piperacillin-tazobactam (2 g/0.25 g) was used and was supplied by Stragen UK (Surrey, United Kingdom). For *in vitro* susceptibility testing, development of drug-containing agar, and high-performance liquid chromatography (HPLC), pure piperacillin-tazobactam was used, which was obtained from Sigma-Aldrich (Dorset, United Kingdom).

### Microorganism.

Pseudomonas aeruginosa (PAO1) was used for all experiments (kindly provided by C. Winstanley [University of Liverpool, United Kingdom]). The bacterium was stored at −80°C in cation-adjusted Mueller-Hinton II (Ca-MH) broth with 10% glycerol (Sigma-Aldrich, Dorset, United Kingdom). For each experiment, fresh isolates were grown on blood agar plates (Oxoid Limited, Hampshire, United Kingdom) at 37°C for 24 h. The frequency of mutation to resistance was estimated, on two separate occasions, by plating aliquots of 0.1 ml of P. aeruginosa (PAO1) onto 10 Ca-MH agar plates containing 24 mg/liter piperacillin and 3 mg/liter of tazobactam (6× the piperacillin MIC). The stability of piperacillin at 4°C was confirmed by quantification of the drug in the agar plates and reproducible enumeration of PAO1 over a 1-week period (data not shown). The bacterial suspension was prepared as for injection into the HFIM (see below). The concentration of the bacterial suspension was determined by quantitative cultures. The ratio of the number of resistant bacteria to the total population was used to estimate the frequency of resistant isolates.

### Susceptibility studies.

The MIC for PAO1 was conducted on five occasions, in Ca-MH broth (Sigma-Aldrich, United Kingdom), using broth microdilution methodology as described by the Clinical Laboratory Sciences Institute (CLSI) (18).

### HPLC.

Piperacillin concentrations were measured using a previously validated high-performance liquid chromatography (HPLC) method with a Shimadzu Prominence system (Shimadzu, Milton Keynes, United Kingdom) (19). Fifty μl of extracted sample was injected onto a Hypersil octyldecyl silane (ODS) C_18_ 5-μm column (150 by 4.6 mm [inside diameter]; Thermo Scientific, Hertfordshire, United Kingdom). A standard curve encompassing 1.56 to 400 mg/liter was constructed in Ca-MH broth, from stock solutions of 8,000 mg/liter piperacillin in water (Fisher Scientific, Loughborough, United Kingdom). The internal standard was penicillin G in water at 1,000 mg/liter (Sigma-Aldrich, Dorset, United Kingdom). The starting mobile phase was 100% A, which was 0.2 M potassium phosphate monobasic buffer in 90:10 water-acetonitrile (vol/vol), with a gradient over 7 min progressing to 50% A and 50% B (acetonitrile) with a run time of 9 min and a flow rate of 1.5 ml/min. Piperacillin was detected using UV at 220 nm. Piperacillin and the internal standard eluted after 6.8 and 7.2 min, respectively. The intra- and interassay coefficient of variation was <5.8%. The limit of detection and quantification was 1.56 mg/liter.

### Hollow-fiber infection model.

**Fig 1:**
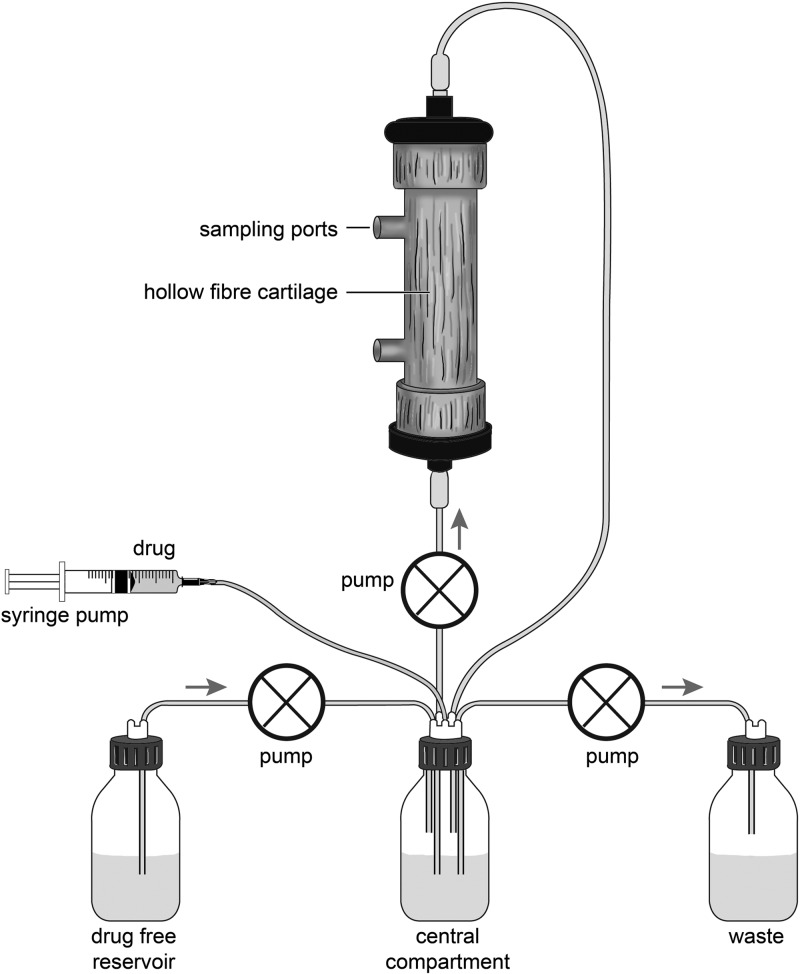
Schematic illustration of the hollow-fiber infection model. The central compartment is connected to the hollow-fiber cartridge, a drug-free reservoir of media, and the waste. Drug may be added to the central compartment via a programmable syringe driver. Courtesy of Helen Carruthers; reproduced with permission.

An HFIM was used to study the response of PAO1 to piperacillin-tazobactam. Two different inocula were used (20). The HFIM circuit is illustrated in Fig. 1. Briefly, outflow from the central compartment (containing 300 ml Ca-MH broth) was connected, via a pump (∼7.2 ml/min) (205 U; Watson-Marlow, United Kingdom), to the hollow-fiber cartridge (FiberCell Systems), which then returned to the central compartment. Fresh Ca-MHB was pumped (∼3.3 ml/min) from a reservoir into the central compartment. Drug was added to the central compartment utilizing a programmable syringe driver (Aladdin pump; World Precision Instruments, United Kingdom). Waste was removed, by pump (∼3.3 ml/min), from the central compartment.

The inoculum was prepared by removing five colonies of PAO1 from the blood agar plate and suspending them in 30 ml Ca-MH broth. The broth was incubated on a shaker at 37°C for 4 h. The optical density of the stock inoculum was adjusted to 0.450 at 600 nm (∼8 × 10^8^ CFU/ml). For the high-inoculum experiments, the stock was used without further dilution. For the low-inoculum experiments, a further 1-in-4,000 dilution was made in Ca-MH broth to achieve a final inoculum of ∼4 ×10^5^ CFU/ml. The high inoculum (∼8 × 10^8^ CFU/ml) was chosen to be comparable to the bacterial density studied in previous *in vitro* studies and, although high, is encountered in patients with VAP (16, 20–22). The lower inoculum (∼4 ×10^5^ CFU/ml) is more typical of patients with VAP. A bacterial density of >10^4^ CFU/ml is considered diagnostic of VAP, while a bacterial density of 10^5^ CFU/ml has been shown to have a greater diagnostic specificity for VAP (23, 24). Each HFIM was then injected, via the sampling port, with 5 ml of either the high or low inoculum. The final inoculum was confirmed by quantitative culture on Ca-MH agar.

### Pharmacokinetic and pharmacodynamic studies.

Piperacillin-tazobactam (2 g/0.25 g) was dissolved in 10 ml sterile 0.9% saline. The study drug was prepared for injection into the circuit in 5-ml syringes following further dilution to achieve the desired concentrations. The effects of various dosages of piperacillin-tazobactam (equivalent to 3 g, 9 g, and 17 g piperacillin in a human) were investigated. Piperacillin-tazobactam was administered over 30 min (bolus) or 4 h (extended infusion) every 8 h for a total of 15 doses. Each experimental run included four HFIMs, with three different piperacillin regimens and a drug-free control. A total of six experiments were performed, including the high inoculum against piperacillin-tazobactam as a bolus (in duplicate), the high inoculum against piperacillin-tazobactam as an extended infusion (in duplicate), the low inoculum against piperacillin-tazobactam as a bolus, and the low inoculum against piperacillin-tazobactam as an extended infusion.

Piperacillin concentrations were estimated by removing 0.5-ml samples from the central compartment at 0.5, 1, 1.5, 2.5, 4.5, and 8 h after the first and seventh dose. Pharmacokinetic samples were stored at −80°C until processing. Drug concentrations were estimated in all HFIM experiments.

The total bacterial density and that of a drug-resistant subpopulation in the HFIM were estimated by plating to drug-free and drug-containing Ca-MH agar plates (Sigma-Aldrich, United Kingdom). Drug-containing Ca-MH agar contained 24 mg/liter piperacillin and 3 mg/liter tazobactam. At 1, 25, 53, 73, 101, and 121 h after inoculation, 0.5 ml was withdrawn from the core compartment of the HFIM. The number of resistant bacteria was defined as the number of organisms enumerated on drug-containing agar. The number of sensitive bacteria was derived from the total number of bacteria counted on drug-free agar minus the number of resistant bacteria.

### Mathematical modeling.

All pharmacokinetic and pharmacodynamic data were comodeled using a population method with the program Pmetrics (version 0.3) (25, 26). The structural mathematical model was based on a previously published model of bacterial resistance (27) and comprised seven inhomogeneous ordinary differential equations.

Equation 1 describes the rate of change of the amount of piperacillin (mg) in the central compartment. A one-compartment model with first-order elimination was used to describe the pharmacokinetic data obtained from all experiments.

Equation 2 describes the rate of change of piperacillin concentrations in an effect compartment.

Equation 3 describes the rate of change of burden of sensitive bacteria in the HFIM, including terms that describe the theoretical maximal bacterial density (equation 3a), the suppression of growth induced by piperacillin (equation 3b), and the rate of bacterial killing induced by piperacillin (equation 3c).

Equation 4 describes the rate of change of bacterial density of resistant bacteria in the HFIM, including terms that describe the theoretical maximal bacterial density (equation 4a) and piperacillin concentration in the effect compartment associated suppression of growth (equation 4b).

Equation 5, 6, and 7 would take the same form as equations 2, 3, and 4 but describe the effect of drug in low-inoculum experiments and are not shown here.

**Fig 2:**
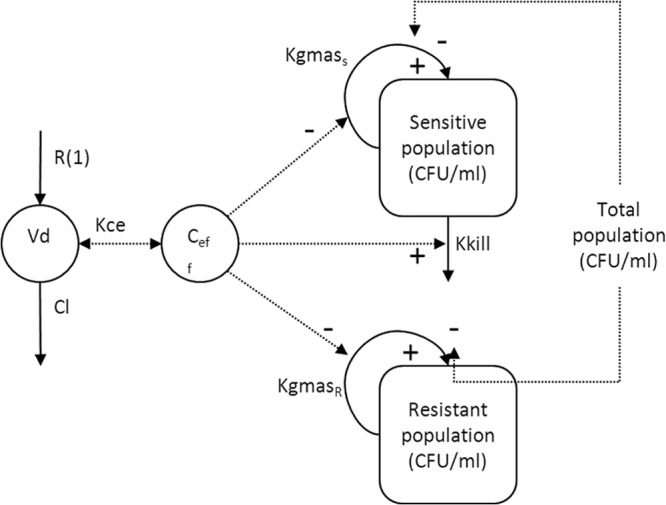
Schematic illustration of the population pharmacokinetic/pharmacodynamic model.

A schematic representation of the structural model is shown in Fig. 2.

$$\frac{dX_{1}}{dt}=R(1)-(\frac{CL}{V_{c}}\times X_{1})$$

$$\frac{dC_{eff}}{dt}=Kce\times (\frac{X_{1}}{V_{c}}-C_{eff})$$

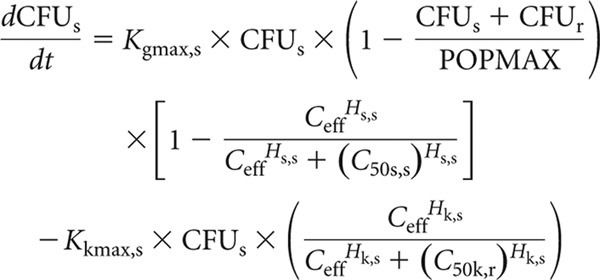


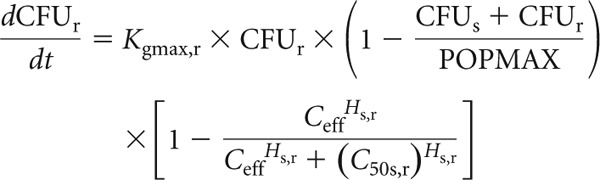



In these equations and in Fig. 2, *X*_1_ is the total amount of piperacillin (mg) in the central compartment. *R*(1) is the infusion of piperacillin into the central compartment. CL is the clearance of piperacillin from the central compartment (liters/h), and *V*_c_ (liters) is the volume of the central compartment. CFU_s_ and CFU_r_ are the number of sensitive and resistant organisms in the HFIM (log_10_ CFU/ml). *K*_ce_ is the rate constant governing movement of piperacillin to and from the central compartment. *K*_gmax,s_ and *K*_gmax,r_ represent the maximum rate of growth of the sensitive and resistant populations, respectively (log_10_ CFU/h). POPMAX (CFU/ml) is the theoretical maximal bacterial burden within the HFIM. *K*_gmax,s_, *K*_gmax,r_ and POPMAX were assumed to be the same in the experiments examining both high and low inocula. *C*_50s,s_ and *C*_50s,r_ (mg/liter) are the drug concentrations that produce half-maximal suppression of growth of the sensitive and resistant populations, respectively. *H*_s,s_ and *H*_s,r_ are the slope functions for suppression of growth of the sensitive and resistant populations. *K*_kmax,s_ (log_10_ CFU/ml) represent the maximum rate of killing of the sensitive population. *C*_50k,s_ (mg/liter) is the drug concentration that produces half-maximal killing of the sensitive and resistant populations, respectively. *H*_k,s_ is the slope function for killing of the sensitive population. Killing of resistant bacteria was not observed and therefore not included in the mathematical model. Clearance, *V*_c_, *K*_gmax,s_, *K*_gmax,r_, and POPMAX were assumed to be the same in both the high- and low-inoculum experiments. All the other pharmacodynamic parameters were specific to the studies examining the high and low inocula.

Error polynomials were obtained by fitting the same structural mathematical model to the pharmacokinetic and pharmacodynamic data from each of the 24 individual HFIMs using the maximum-likelihood estimator in the program ADAPT 5 (28, 29). The means, medians, and standard deviations of the population parameters were estimated. The fit of each of the structural models to the data was further assessed by (i) the log likelihood value, (ii) the coefficient of determination (*r*^*2*^) from a linear regression of the observed- predicted plots both before and after the Bayesian step, (iii) the Akaike information criterion, and (iv) comparison of the observed data with simulation based on the mean and median population parameter estimates (1).

### Pharmacokinetic and pharmacodynamic simulations.

Simulations were performed for each regimen and inoculum, using the mathematical model described above. The pharmacokinetic program ADAPT 5 was used (28). Both the population and the individual median parameter estimates (i.e., after the Bayesian step) for each of the 24 HFIMs were used. A number of endpoints were explored and reported in the simulations. The fraction of the final 3 dosing intervals (i.e., the final 24 h of the experiment, on day 5) during which the drug concentrations were above the MIC and 4 times the MIC and the area under the piperacillin concentration-time curve were estimated (30).

A second set of simulations was performed for 17 piperacillin regimens between 0 and 20 g three times daily, administered by bolus or extended infusion. The median parameter estimates for the population were used. The density of total bacteria and the resistant subpopulations after 121 h (15 doses) and trough piperacillin concentration were estimated. The trough piperacillin concentration was adjusted for MIC (*C*_min_/MIC ratio).

### Extrapolating from the HFIM to humans.

To explore the clinical implication of the experimental observations, the results obtained with the HFIM were extrapolated to humans. A previously published parallel first-order Michaelis-Menten clearance model for piperacillin plasma pharmacokinetics was used (13). Four regimens were investigated using a 5,000-patient Monte Carlo simulation. The regimens were (i) 4 g piperacillin administered over 30 min with repeat dosing every 8 h, (ii) 4 g piperacillin administered over 4 h with repeat dosing every 8 h, (iii) 4 g piperacillin administered over 30 min with repeat dosing every 6 h, and (iv) 4 g piperacillin administered every 3 h with repeat dosing every 6 h. The mean parameter values and their associated variance (obtained from the output of the original population pharmacokinetic model (13) were embedded in the subroutine PRIOR of the ADAPT 5 program (28). The parameter estimates ± standard deviations were as follows: *V*_max_, 898.91 ± 402.61 mg/h; *K*_m_, 90.13 ± 74.14 mg/liter; *V*_c_, 13.67 ± 7.20 liters; *K*_cp_, 9.19 ± 10.25 h^−1^; *K*_pc_, 20.95 ± 16.91 h^−1^; and SCL, 6.62 ± 3.81 liters/h (13). A log-normal parameter distribution was used in the simulations. Protein binding for piperacillin was assumed to be 30% (31). For each regimen, the fraction of simulated subjects who achieved the pharmacodynamic target of a trough total piperacillin concentration/MIC ratio of either 3.4 for bolus regimens or 10.4 for extended infusions, for a range of MICs from 0.0625 to 64 mg/liter, was determined.

## RESULTS

### MICs.

The piperacillin MICs in Ca-MH broth for PAO1 were 2, 4, 4, 8, and 8 mg/liter. A median MIC of 4 mg/liter was used in subsequent analyses.

### Mutational frequency to resistance.

The frequency of mutation of PAO1 to piperacillin resistance was 1.04 × 10^−7^ at 6 times the MIC (i.e., there was 1 resistant bacterium for every 1.04 × 10^7^ bacteria).

### Results of the hollow-fiber infection model. (i) Pharmacokinetics.

**Fig 3:**
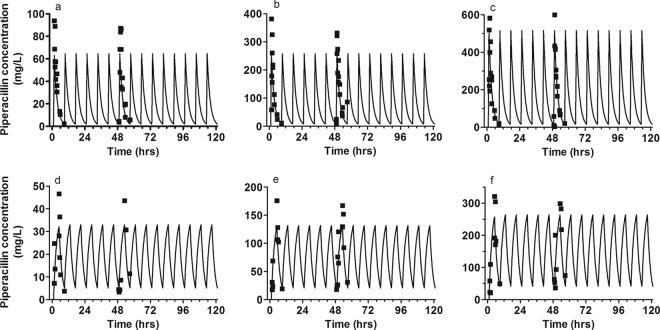
Pharmacokinetic data. The fit of the mathematical model to the observed piperacillin concentrations for 3-g, 9-g, and 17-g boluses (a to c) and 3-g, 9-g, and 17-g extended infusions (d to f) is shown.

Extended-infusion regimens maintained the piperacillin concentration above the MIC for a greater proportion of the dosing interval. Trough piperacillin concentrations were lower with the bolus regimen than with the same total dosage delivered by extended infusion (Fig. 3; Table 1 and 2).

**Table 1:** Parameter estimates from the mathematical model

Inoculum	Mean ± SD (median)														
	CL (liters/h)	*V* (liters)	POPMAX (log_10_ CFU/ml)	*K*_gmax,s_ (log_10_ CFU/ml/h)	*K*_gmax,r_ (log_10_ CFU/ml/h)	*K*_ce_ (h^−1^)	*C*_50s,s_ (mg/liters)	*H*_s,s_	*K*_kmax,s_ (log_10_ CFU/ml/h)	*H*_k,s_	*C*_50k,s_ (mg/liter)	*C*_50s,r_ (mg/liter)	*H*_s,r_	Initial conditions (total)	Initial conditions (resistant)
High and low	0.167 ± 0.037 (0.164)	0.367 ± 0.073 (0.355)	3.89 × 10^9^ ± 2.91 × 10^9^ (3.25 ×10^9^)	0.642 ± 0.035 (0.630)	0.450 ± 0.044 (0.445)										
High						0.064 ± 0.015 (0.061)	2.805 ± 1.588 (3.458)	8.364 ± 2.734 (9.792)	0.086 ± 0.017 (0.097)	2.089 ± 2.854 (1.197)	343.664 ± 302.027 (231.662)	35.516 ± 4.334 (36.298)	1.986 ± 2.480 (0.978)	3.51 ×10^7^ ± 1.61 ×10^7^ (3.20 ×10^7^)	11.998 ± 6.929 (8.554)
Low						0.056 ± 0.018 (0.060)	3.189 ± 1.211 (3.093)	2.510 ± 1.644 (1.670)	0.075 ± 0.029 (0.092)	3.321 ± 1.561 (3.843)	72.279 ± 16.455 (73.011)	32.435 ± 7.323 (33.819)	10.346 ± 2.218 (10.862)	5.34 ×10^4^ ± 2.64 ×10^4^ (5.11 ×10^4^)	0.038 ± 0.011 (0.036)

**Table 2:** Selected pharmacokinetic data for piperacillin^*a*^

Piperacillin dosage and regimen	*T* > MIC (%)	*T* > 4× MIC (%)	AUC (mg/liter/24 h)	(mg/liter)
3-g bolus	81	43	457.8	2.0
3-g extended infusion	100	59	457.8	5.2
9-g bolus	100	81	1,831.3	8.1
9-g extended infusion	100	100	1,831.3	20.8
17-g bolus	100	100	3,662.5	16.2
17-g extended infusion	100	100	3,662.5	41.6

a *T* > MIC and *T* > 4× MIC, time the free piperacillin concentration in the central compartment was above the MIC and above four times the MIC.

### (ii) Untreated controls at the high and the low inoculum.

**Fig 4:**
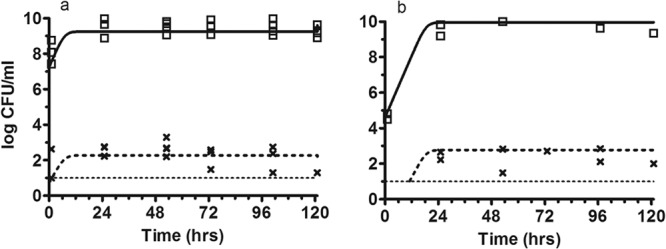
Control data. Observed bacterial densities for the total (□) and resistant bacterial (×) populations are shown. The solid and dotted lines represent the fit of the mathematical model to the data. (a) High inoculum, controls; (b) low inoculum, controls.

Logarithmic growth occurred in the total bacterial population with an estimated maximum bacterial density of 3.89 × 10^9^ CFU/ml (Table 1; Fig. 4). Growth of the resistant subpopulation achieved a maximum density of ∼10^2^ CFU/ml, at which point there was no further growth of the resistant subpopulation. The final bacterial densities of both the total population (POPMAX) and the resistant subpopulation were similar for both inocula.

### (iii) Impact of a bolus regimen and extended-infusion regimen of piperacillin to treat a high inoculum of PAO1.

**Fig 5:**
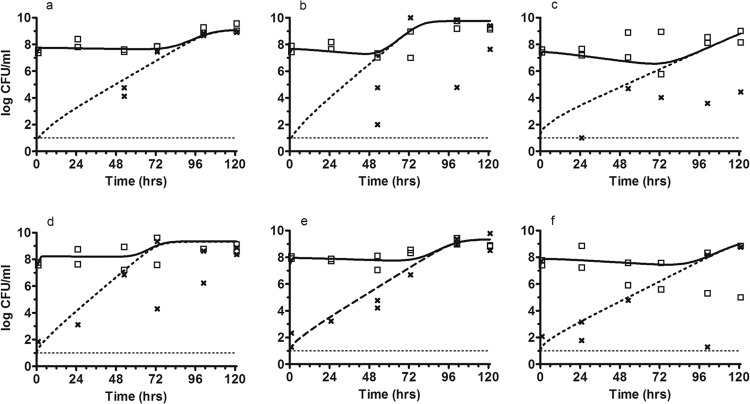
Effect of piperacillin-tazobactam therapy with a high inoculum. The observed bacteria densities for the total (□) and resistant (x) bacterial populations are shown. The solid and dotted lines represents the fit of the mathematical model to the data for 3-g, 9-g, and 17-g boluses (a to c) and 3-g, 9-g, and 17-g extended infusions (d to f).

Suppression of total bacterial growth was observed in the initial 48 to 72 h with all dosages (Table 1 and Fig. 5). Amplification of a resistant subpopulation occurred with all piperacillin dosages administered as either a bolus or an extended infusion (Fig. 5). Dose-dependent suppression of growth of the resistant subpopulation was seen. For all three dosages, the total bacterial population was replaced by the resistant subpopulation by 96 h. For the highest dosages, there was suppression of growth of the total bacterial population, but the population consisted entirely of a resistant subpopulation. Expansion of the resistant subpopulation occurred at similar rates following administration of piperacillin by bolus dosing and by extended-infusion regimens.

### (iv) Impact of the bolus regimen and the extended-infusion regimen of piperacillin to treat a low inoculum of PAO1.

**Fig 6:**
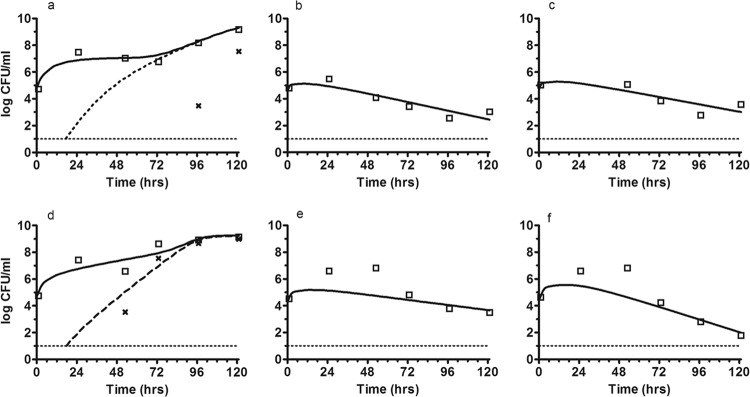
Effect of piperacillin-tazobactam therapy with a low inoculum. The observed bacteria densities for the total (□) and resistant (x) bacterial populations are shown. The solid and dotted lines represents the fit of the mathematical model to the data for 3-g, 9-g, and 17-g boluses (a to c) and 3-g, 9-g, and 17-g extended infusions (d to f).

At the lowest dosage, suppression of growth of the total bacterial population was observed (Table 1; Fig. 6). However, there was progressive growth of the resistant subpopulation. By 120 h, all bacterial isolates were resistant to piperacillin. Following the administration of 9 g and 17 g of piperacillin, there were reductions in total CFU of ∼3.6 and ∼1.4 log_10_ CFU/ml for the bolus regimen and ∼3.4 and ∼1.1 log_10_CFU/ml for the extended infusion, respectively. No resistance emerged at the highest two dosages of piperacillin.

### Mathematical modeling.

The fit of the mathematical model to the observed data was acceptable. The *r*^*2*^ for the observed versus predicted piperacillin concentrations was 0.908 using the median model parameter values (Bayesian prior) to calculate predicted values. For the high-inoculum experiments, the *r*^*2*^ values of Bayesian prior predictions versus observations for the bacterial densities were 0.329 and 0.782 for the total population and resistant subpopulation, respectively. For the low-inoculum experiments, the *r*^*2*^ values of Bayesian prior predictions for the bacterial densities were 0.884 and 0.885 for the total population and resistant subpopulation, respectively. The *r*^*2*^ for the linear regression for all observed-predicted values improved by using the median parameter values of the Bayesian posterior obtained by updating the prior distribution for each experiment. Faster growth occurred in the sensitive subpopulation than the resistant subpopulation (i.e., *K*_gmax,s_ of 0.642 log_10_ CFU/ml/h versus *K*_gmax,r_ of 0.450 log_10_ CFU/ml/h).

### Bridging study.

**Fig 7:**
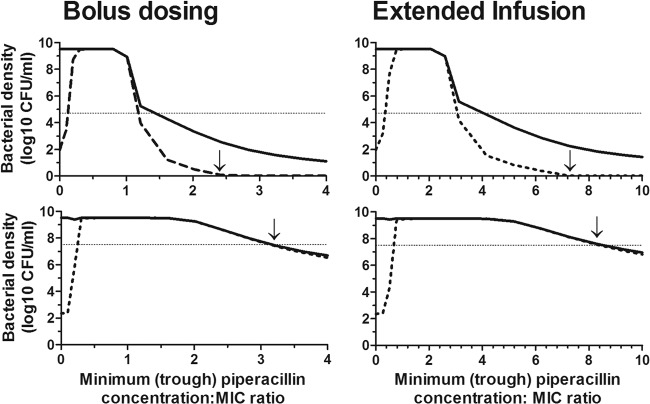
Change in bacterial density with the trough free piperacillin/MIC ratio following 5 days of treatment and target attainment of clinical regimens. Solid line, total population; dashed line, resistant subpopulation; dotted line, stasis line. The arrow indicates the relevant *C*_min_/MIC ratio.

The simulations suggested a dose-dependent change in bacterial densities based on findings from the high- and low-bacterial-inoculum experiments (Fig. 7). The changes in bacterial densities with increasing piperacillin exposure are represented by an inverted U-shaped curve similar to that described for fluoroquinolones (17). At high bacterial densities (∼7 log_10_CFU/ml), trough plasma piperacillin concentration/MIC ratios of 4.6 and 11.9, for the bolus and extended-infusion regimens, respectively, were required to suppress growth of the total bacterial population (Table 3). However, at these trough concentrations there was expansion of the resistant subpopulation.

**Table 3:** *C*_min_/MIC ratios required to achieve stasis, 1-, 2-, and 3-log bacterial killing and suppression of emergence of resistance

Bacterial density and status	*C*_min_/MIC (mg/liter)			
	Bolus		Extended infusion	
	Hollow fiber	Predicted plasma^*a*^	Hollow fiber	Predicted plasma^*a*^
Low				
Bacterial stasis (total bacteria)	1.4	2.0	4.1	5.9
1-log reduction in total CFU/ml	1.8	2.6	5.2	7.4
2-log reduction in total CFU/ml	2.4	3.4	6.7	9.6
3-log reduction in total CFU/ml	3.2	4.6	8.8	12.6
Suppression of resistance	2.4	3.4	7.3	10.4
High				
Bacterial stasis (total bacteria)	3.2	4.6	8.3	11.9

a Protein binding is assumed to be 30% (31).

**Fig 8:**
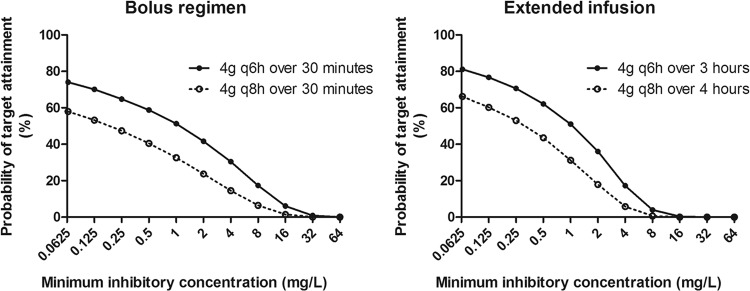
Results of the Monte Carlo simulation with the probability of target attainments against a range of MICs for the following regimens: 4 g piperacillin administered intravenously (i.v.) for either 30 min or 4 h every 8 h as well as for 30 min or 3 h every 6 h.

At low initial bacterial densities, trough plasma piperacillin concentration/MIC ratios of 3.4 and 10.4 for the bolus and extended-infusion regimens, respectively, were required to suppress bacterial resistance. At these trough concentrations, there was a >2-log reduction in total bacterial density. The Monte Carlo simulation showed that ∼60% of patients administered the bolus or extended-infusion regimens were expected to achieve these trough concentrations with a regimen of 4 g administered every 8 hours when the MIC was low (Fig. 8). Increase in the MIC results in a reduction of the probability of target attainment, with suppression of resistance occurring in 14.6% and 5.8% of patients when the MIC is 4 mg/liter. Administration of 16 g of piperacillin daily improved the target attainments to ∼80% for either regimen for highly susceptible organisms.

## DISCUSSION

This study investigated the impact on antibacterial activity and the emergence of piperacillin resistance following administration of piperacillin-tazobactam by bolus versus extended infusion. Piperacillin administration by bolus and that by extended infusion result in comparable antibacterial activities and rates of emergence of antimicrobial resistance. In the setting of a high bacterial inoculum, expansion of a resistant subpopulation occurs even when there is an overall decline in bacterial density. In a clinical setting, a decline in bacterial density may be achieved only at the expense of the development of a resistant bacterial subpopulation. These resistant bacteria may be responsible for a subsequent clinical relapse in an individual patient or serve as a source for horizontal transmission. Prevention of the emergence of a resistant subpopulation in the setting of a high bacterial inoculum may require an alternative therapeutic approach, such as combination chemotherapy. In the low-bacterial-inoculum experiments, trough plasma total piperacillin concentration/MIC ratios of 3.4 and 10.4, from the bolus and extended-infusion regimens, respectively, were required to suppress the emergence of antimicrobial resistance. Results of the Monte Carlo simulation suggest that that fewer than 11% of patients achieve these concentrations when regimens of piperacillin of 12 g/day in three divided dosages for an organism with an MIC of 4 mg/liter are used. Although treatment of organisms with lower MICs or increasing the total piperacillin dosage to 16 g daily leads to an increase the rates of target attainment, this is still far from optimal.

A bacterial density of >10^4^ CFU/ml in bronchoalveolar lavage fluid is required for a diagnosis of VAP (23, 32). However, bacterial densities as high as 10^8^ CFU/ml are frequently encountered in patients with this syndrome (21). Bacterial densities spanning a similar range (10^4^ to 10^8^ CFU/ml) are also present in patients with HAP (33). The high and low inocula used in this *in vitro* study were selected to encompass the range of these clinically relevant bacterial densities. Antimicrobial management for patients with HAP and VAP is complex, with many conflicting results from both clinical and preclinical studies (23). *In vitro* studies, including this study, suggest that monotherapy with a β-lactam agent to treat high densities of Pseudomonas aeruginosa is insufficient to suppress the emergence of antimicrobial resistance (30, 34). The addition of a second agent to a β-lactam (e.g., an aminoglycoside or fluoroquinolone) may enable suppression of emergence of resistance, but this requires further study (35).

The pharmacokinetic variability of β-lactam antibiotics in critically ill patients has led to suggestions that therapeutic drug monitoring may be a useful adjunct to therapy (36). Trough concentrations are a clinically convenient therapeutic target because of the ease with which samples can be interpreted. The fraction of the dosing interval during which drug concentrations are above a threshold (e.g., some multiple of the MIC) requires more intensive sampling. When the bacterial burden is ∼10^4^ CFU/ml (the density required for a diagnosis of VAP), the piperacillin concentration/MIC ratios within the HFIM that suppresses the emergence of piperacillin resistance are 2.4 and 7.3 for the bolus and extended-infusion regimens, respectively. This corresponds to plasma *C*_min_/MIC ratios of 3.4 and 10.4 for the bolus and extended-infusion regimens, respectively, assuming that piperacillin protein binding is 30%. The *C*_min_/MIC ratio is consistent with the ratio of 6.2 for bolus regimens of meropenem (16). The *C*_min_/MIC ratios identified in the HFIM are achieved in insufficient numbers of critically ill patients, suggesting that considerable dosage escalation may be required to achieve adequate drug exposure.

A limitation of the HFIM is the lack of immune-mediated bacterial killing. Nevertheless, the absence of immune function permits the direct estimation of the extent of antimicrobial activity that can be attributed to a drug. The pharmacodynamic targets identified in the HFIM may be different if immune-mediated bacterial killing is also present. This provides a safety margin when the results are extrapolated to humans by delineating a “worst-case scenario.” Additionally, the results from the HFIM may be applied to immunocompromised patients. The pharmacodynamic targets identified with the HFIM require validation with additional strains of Pseudomonas with different MICs and/or mechanisms of antimicrobial resistance.

In summary, this study suggests the following: (i) bolus regimens are equivalent to intermittent infusion in terms of the antibacterial effect and the emergence of drug resistance, and (ii) bacterial burden has a significant influence on the ultimate outcome of antibacterial therapy. Patients with low bacterial burdens (e.g., ∼10^4^ CFU/ml) of Pseudomonas aeruginosa may potentially be treated with monotherapy, with little chance of driving resistance. This will minimize the potential adverse events associated with combination chemotherapy, such as nephrotoxicity from aminoglycosides. In contrast, patients with a higher bacterial density may require additional adjunctive therapies, such as combination chemotherapy, to prevent the emergence of antimicrobial resistance. The impact on bacterial density on emergence of antimicrobial resistance in other bacterial species warrants further *in vitro* investigation. Future clinical management of patients with HAP and VAP may require patients to be stratified according to the antimicrobial resistance pattern of the bacterial species and pathogen density in order to select the optimal individual regimen.
